# Exposure to intimate partner violence reduces the protective effect that women’s high education has on children’s corporal punishment: a population-based study

**DOI:** 10.3402/gha.v7.24774

**Published:** 2014-09-12

**Authors:** Mariano Salazar, Kjerstin Dahlblom, Lucia Solórzano, Andrés Herrera

**Affiliations:** 1 Epidemiology and Global Health, Department of Public Health and Clinical Medicine, Umeå University, Sweden; 2 Center for Demography and Health Research, Nicaraguan National Autonomous University, León, Nicaragua

**Keywords:** children's corporal punishment, education, interaction, IPV, women

## Abstract

**Background:**

Previous studies have shown that women's education is protective against corporal punishment (CP) of children. However, the effect that women's exposure to intimate partner violence (IPV) has on the association between women's education and children's CP has not been studied.

**Objective:**

To understand how the interaction between women's exposure to IPV and their education level influences the occurrence of children's CP at the household level.

**Methods:**

We selected 10,156 women who had at least one child less than 16 years old from cross-sectional data from the 2006–2007 Nicaraguan Demographic and Health Survey. Children's CP was defined as the punishment of children by slapping them, hitting them with a fist, or hitting them with a rope, belt, stick, or other object. IPV was measured by using a conflict tactic scale. The WHO Self-Reporting Questionnaire 20 (SRQ-20) was used to assess the women's mental health. We computed adjusted risk ratios (ARR) and 95% confidence intervals (CI) using Poisson regression with a robust variance estimator.

**Results:**

Women's exposure to IPV was associated with a 10–17% increase in the risk of children's CP. IPV and children's CP were associated with impaired women's mental health. Women's lifetime exposure to emotional IPV and controlling behavior by a partner significantly decreased the protective effect from women's high education level on children's CP. When women were exposed to emotional IPV, the protective effect from having a college education decreased from ARR=0.61 (95% CI 0.47–0.80) to ARR=0.98 (95% CI 0.80–1.19). A similar pattern was found among women exposed to controlling behavior by a partner, the protective effect decreased from ARR=0.71 (95% CI 0.53–0.90) to ARR=0.86 (95% CI 0.70–1.06).

**Conclusion:**

This study shows how significant gains in one positive social determinant of children's well-being can be undermined when it interacts with men's violence toward women. Policies that aim to end children's CP must include actions to end women's exposure to IPV.

The use of corporal punishment (CP) to discipline children is a highly controversial issue around the world. It has been criticized as being a consequence of the parent's frustration with the child's behavior and as being based on dominance and control instead of a more horizontal approach (1, 2). The United Nations has recommended the elimination of all forms of children's CP (3) because such punishments have been shown to have a negative influence on the children's current and future health (2, 4–7). In spite of the negative effects on children's mental and physical health, children's CP is still a legal, widespread, and culturally accepted social practice in many places (8). In the international arena, countries banning children's corporal punishment are few in comparison to those allowing it (24 vs. 171), and those prohibiting the practice are mainly located in Europe (8).

Corporal punishment affects children of all ages, with usage reaching a peak with toddlers and decreasing in adolescence (9). In the United States, Zolotor et al. analyzed retrospective data from a population-based surveys and found that rates of moderate children's CP (slapping/spanking a child) decreased by 18% from 1972 to 2002. Nevertheless, in 2002, the rates of moderate children's CP were 788 per 1,000 for children aged 3–5 years, 600 per 1,000 for children aged 6–8 years, and 524 per 1,000 for children aged 9–11 years (8). In the same year, rates of severe children's CP (defined as hitting a child with an object) were lower than for moderate children's CP, but the data still showed that 3 out of 10 children aged 3–11 years were punished in this way in the United States (8).

Children's CP is also common in low- and middle-income countries. Akmatov (10) analyzed population-based data from 28 low- and middle-income countries and found that the 12-month prevalence of moderate children's CP ranged from 20 to 81%. Severe children's CP was less common – but still high in some settings – and ranged from 2 to 61% (10). Population-based data also showed that Latin American children are frequently exposed to CP (11–13). For example, one out of five Brazilian children had experienced severe CP in the past year (11). In Peru and Chile, 19 and 37% of mothers, respectively, reported that they discipline their children by using physical violence (12, 13).

## Factors associated with children's CP

Children's CP is a complex phenomenon influenced by factors at different levels in society. Strauss developed an ecological conceptual framework that identifies proximal, mezzo, and distal factors linked with higher children's CP rates (9). The proximal factors involve the caregiver and household characteristics. The mezzo factors cluster around legal aspects and cultural beliefs, and the distal factors focus on inequalities in societies (9).

In both low- and high-income countries, important proximal factors that increase the occurrence of children's CP include low socioeconomic status, caregivers’ unemployment (10, 14, 15), young caregivers (15), large family size (7, 10), poor maternal mental health (16–18), and rural residency (7). Maternal education stands as one of the most important proximal protective factors for children's CP. Several studies have found that the prevalence of children's CP increases as maternal education decreases and vice versa (7, 14, 16, 19). Violence against children cannot be separated from the violence that their caregivers experience. Traditionally, patriarchal gender relations have imposed the task of raising children mainly on women. Thus, it is not surprising that women's exposure to one of the most common forms of violence against women, intimate partner violence (IPV) (20), has been consistently associated with higher rates of children's CP around the world (12, 16, 21–23). For example, one study conducted in the United States found that women exposed to physical IPV by their current partner had 1.49 times higher odds of spanking their children than women not exposed to IPV, even after adjusting for possible confounding factors (16).

Cultural beliefs figure among the most important mezzo factors that influence children's CP, and children's CP has been shown to be more common in settings where caregivers have a normalized perception of physical punishment (10, 17, 24–26). Cappa and Khan (26) analyzed data from 34 household surveys from low- and middle-income countries and found that in almost all of those countries, caregivers’ approval of physical punishment was strongly associated with children's CP. They also found that norms justifying children's CP were closely correlated to norms tolerating IPV (26). A country's legal environment is another mezzo factor that influences parents’ attitudes toward children's CP. Parents who live in countries where children's CP is illegal exhibit stronger attitudes in support of ending children's CP than do parents who live in countries where no ban against children's CP exists (8). However, data are inconclusive on whether children's CP diminishes in countries that legally ban CP (8). For example, children's CP rates have diminished in Sweden after the ban, but an increase in cases reported to the police has been found in other countries with an active ban such as Norway and Romania (8).

Studies on the distal factors that influence children's CP are scarce. One example is an ecological study conducted by Ember and Ember that used ethnographic data from 186 pre-industrial societies (27). Ember and Ember found that children's CP was more frequent in those societies that exhibited high levels of social inequalities and in those where the indigenous populations were ruled by a foreign power (27).

## Rationale

In the past 40 years, women's education levels have experienced a significant increase worldwide, rising from a mean of 2.2 years of education in 1970 to 7.2 years in 2009 (28). This significant improvement in women's education levels has proven to be essential for children's health and well-being. For example, studies analyzing pooled data from all over the world have shown that high maternal education level is strongly linked to lower morbidity (29) and mortality (28) of children. It has been theorized that these effects are mediated by the positive effects that high education levels have on women's personal autonomy and economic position in society, among other pathways (28). Population-based studies have also shown that women often experience violence by their current or former partners (20, 30), and this violence has been reported to have a detrimental effect on the well-being of women (31) and their children (32–34). As described above, both women's educational attainment (7, 9, 14, 16, 19) and women's exposure to IPV (12, 16, 21–23) have been linked to children's CP. However, how the rates of children's CP vary when these two fundamental social determinants interact has not yet been studied. We hypothesized that women's exposure to different forms of IPV reduces the protective effect that women's higher education level has on children's CP. We believe that understanding the interaction between exposure to different types of IPV and women's education level will provide useful evidence for stakeholders and policy makers to design and implement evidence-based programs to reduce the occurrence of children's CP around the world.

## Materials and methods

### Setting

This study was conducted in Nicaragua, a low-income country located in the middle of the American continent. As in many other low-income countries, Nicaraguan women have experienced an increase in their mean years of education, which have risen from 1.9 years in 1970 to 5.8 years in 2009 (28). In spite of this, Nicaraguan women are often exposed to different forms of IPV, and data from the 2006–2007 nationwide demographic and health survey (DHS) indicated that lifetime exposure to IPV varied from 56% for controlling behavior by a partner to 15% for sexual IPV (35). Lifetime physical abuse by a partner was reported by 3 out of 10 women interviewed, and lifetime emotional abuse was reported by one out of two women (35). Exposure to IPV is far from being an occasional incident. A panel study in this setting found that 23% of the women interviewed experienced a continued pattern of abuse spanning for at least four years (30).

Children's CP is common, socially accepted, and legal in Nicaragua. The legal provisions allow CP of children at home but not in schools (36). A population-based study conducted in an urban and rural setting found that 7 out of 10 adults interviewed reported that children living in their households experience some kind of CP when they misbehave, and when they do, they are punished frequently and mostly by their mothers (37). This study also showed that children's CP is endorsed by most adults, with higher endorsement in rural areas than in urban settings (52 and 37%, respectively) (37).

### Study design

This study used cross-sectional data collected for the 2006–2007 DHS conducted by the Nicaraguan National Institute for Information Development (35). The DHS assessed women's demographic and reproductive characteristics and provided demographic information about the women's households. The data were collected through multistage cluster sampling, and only one woman per household was interviewed. A detailed sample design and data collection strategy can be found elsewhere (35). For the current study, we selected 10,156 women who reported having at least one child less than 16 years of age.

### Measurements

The outcome measure (children's CP) was assessed using the following question: ‘In this household, how are children punished when they misbehave?’ Children's CP was defined as the punishment of children by slapping them, hitting them with a fist, or hitting them with a rope, belt, stick, or other object. The variable constructed had two options: yes (1) and no (0). Yes, meant that the children were exposed to any of the forms of CP described above, and no meant that the children were not exposed to CP. The survey asked about the women's attitudes toward CP with the question, ‘Do you believe that physical punishment is needed to educate children?’ Answers were recorded as yes or no.

Exposure to emotional, physical, and sexual IPV and to controlling behavior by a woman's current or former partner was assessed using questions from the WHO Multi-Country Study on Women's Health and Domestic Violence (20). This WHO instrument has been validated in multiple settings, including Nicaragua, and has shown good internal consistency and the capacity to discriminate between different forms of IPV (20, 30). Yelling, humiliation, intimidation, and threats were considered emotional IPV. Acts labeled as physical IPV ranged from slaps, pushes, punches, kicks, and strangulation to the use of weapons. Sexual IPV was defined as being physically forced to have intercourse or accepting to have intercourse due to fear of the partner's reaction. The following actions by a partner were labeled as controlling behavior: limiting a woman's contact with family, friends, or healthcare professionals; insisting on knowing where a woman is at all times; getting upset if a woman talks to other men; or always suspecting a woman of infidelity. A woman was considered to have been exposed to an IPV category if she answered yes to any of the items described within that category.

Women's education was measured directly by asking the respondents what was the highest education level that they had attained. This was later summarized into the following four groups: no education, primary school education, high school education, and college education. The household socioeconomic status was used as a proxy for the women's socioeconomic status. This was measured with a household assets index based on whether the household had several items including electronic devices and forms of transportation. Principal component analysis was used to create a total household index that was divided into quintiles (35).

The WHO Self-Reporting Questionnaire 20 (SRQ-20) (38) was used to measure women's mental health. The SRQ-20 is a screening tool that contains 20 questions assessing different symptoms of mental health impairment. It has been validated in Nicaragua and elsewhere (38, 39). Each affirmative answer to a question was coded as 1, and negative answers were coded as 0. All answers were added together to create an overall score. Other variables collected in the survey were women's residency (urban, rural), age (years), and parity.

### Analysis

We used STATA 12 (StataCorp, College Station, Texas) survey command (svy) to analyze the data. It allows the use of sampling weights in all analyses to compensate for the women's unequal selection probability due to the sampling method used to collect the data. Also, it allows the use of robust variance estimators to calculate standard errors (40). Data were described using weighted means and percentages. Differences between groups were assessed using Student's *t*-test to compare means across groups and the chi-squared test to compare proportions.

For the multivariable analyses, we used Poisson regression with a robust variance estimator to compute adjusted risk ratios (ARR) and 95% confidence intervals (CI). This method was used instead of logistic regression because the prevalence of the outcome was higher than 10%. In this case, the odds ratios could overestimate the relative risk between the variables under study (41, 42). The Poisson analyses were completed for each type of IPV exposure with two models: Model 1 described the adjusted association between IPV, women's education level, and children's CP, and Model 2 included an interaction term for women's education level and IPV exposure. The association between IPV exposure and children's CP was further stratified by women's education level for the models that showed significant multiplicative interaction (*p*<0.05).

A variable was considered a confounder and was included in the final models if it was associated with both IPV exposure and the outcome. We used a cutoff value of *p*<0.20 to include a variable in the models. Thus, the analyses were adjusted by the women's age, education level, residency, socioeconomic status, parity, and attitude toward children's CP. Women's mental health scores (from the SRQ-20) were considered to be an intermediate factor between IPV exposure and the occurrence of children's CP and, therefore, were excluded from the multivariable analyses. We assessed multicollinearity between the variables included in the models using tolerance and variance inflation factor (VIF) diagnostic tests. Multicollinearity was defined as tolerance values below 0.1 and VIF values equal to or greater than 10 (43). No multicollinearity between the variables was found. Associations were considered significant if *p*<0.05.

### Ethical considerations

The current study is a re-analysis of secondary data collected by the Nicaraguan government as part of the 2006–2007 nationwide DHS. The dataset used is open access and can be downloaded from http://ghdx.healthmetricsandevaluation.org/record/nicaragua-reproductive-health-survey-2006–2007. The study complies with the Helsinki Declaration. The dataset does not contain individual or household data (names, identification numbers, or addresses) that can be used to identify the women or the children included in this study.

## Results

### Prevalence and characteristics of CP used with children

Children's exposure to CP was common in Nicaragua. Thirty-four percent of the women reported that misbehaving children were disciplined using some form of CP. Among the women reporting that CP was used in the household, almost all (91%) reported that children were disciplined by hitting them with a belt, ruler, rope, or other objects. At the bivariate level, children's exposure to CP was associated with the following characteristics of the women: rural residency, lower education level, lower socioeconomic status, higher parity, higher SRQ-20 scores, approval of CP, and having a partner at the time of data collection (Table 1, *p*<0.05).

**Table 1:** Women's characteristics stratified by usage of children's corporal punishment

Women's characteristics	Corporal punishment No (n=6,587)	Corporal punishment Yes (n=3,569)
Age in years, mean (SE)†	30.6 (0.15)	31.1 (0.18)
Women's education*		
No education	13.0%	22.0%
Primary school	39.7%	45.4%
High school	34.5%	24.9%
College	12.8%	7.7%
Total	100.0%	100.0%
Residency (rural)*	41.3%	53.2%
Parity, mean (SE)*	2.8 (0.04)	3.6 (0.06)
Household socioeconomic status*		
Low	18.8%	29.1%
Medium-low	20.0%	22.7%
Intermediate	20.6%	20.3%
Medium-high	20.8%	16.0%
High	19.8%	11.9%
Total	100.0%	100.0%
Approves of corporal punishment*	3.3%	33.9%
SRQ-20 score, mean (SE)*	4.9 (0.08)	5.8 (0.12)
Marital status (currently partnered)*	73.3%	77.3%

Weighted means, standard errors (SE), and percentages are shown (n=10,156).

* *p*<0.05, chi-squared or Student's *t*-test

† *p*<0.06.

### IPV exposure and children's CP

Women's lifetime exposure to IPV was common, with exposure to controlling behavior by a partner (52.8%) and emotional abuse (46.5%) occurring the most frequently (Table 2, *p*<0.05). Bivariate analyses showed that all forms of IPV (emotional, physical, sexual, and controlling behavior) were significantly associated with higher prevalence of children's CP (Table 2, *p*<0.05) and impaired women's mental health as represented by higher mean scores on the SRQ-20 (data not shown).

**Table 2:** Women's exposure to different forms of IPV stratified by children's exposure to corporal punishment

Type of exposure	All (n=10,156)	Corporal punishment No (n=6,587)	Corporal punishment Yes (n=3,569)
Emotional IPV	46.5%	43.6%	52.2%*
Physical IPV	26.5%	23.6%	32.1%*
Sexual IPV	12.8%	11.5%	15.5%*
Controlling behavior by a partner	52.8%	49.9%	58.5%*

Weighted percentages are shown.

* *p*<0.05, chi-square.

IPV=intimate partner violence.

### Multivariable analysis

The multivariable analyses showed that all forms of IPV were associated with higher risk of children's CP, ranging from a 10% increased risk for CP associated with lifetime exposure to sexual IPV to a 17% increased risk associated with lifetime exposure to emotional IPV (Table 3, Model 1, *p*<0.05). Model 1 also showed that for all types of IPV, the risk of children's CP decreased as women's education level increased (Table 3, *p*<0.05). Lifetime exposure to emotional abuse and lifetime exposure to controlling behavior by a partner showed significant interactions with women's education level (Table 3, Model 2, *p*<0.05). The associations between children's CP and women's education level were further stratified by women's exposure to lifetime emotional IPV and controlling behavior by a partner. The stratification showed heterogeneity of effects. Exposure to lifetime emotional IPV or controlling behavior by a partner significantly decreased the protective effect that women's higher educational levels had on children's CP (Table 4, *p*<0.05). This effect was more pronounced among women with high school or college education. For example, when women were exposed to emotional IPV, the protective effect from having a college education decreased from ARR=0.61 (95% CI 0.47–0.80) to ARR=0.98 (95% CI 0.80–1.19). A similar pattern was found among women exposed to controlling behavior by a partner, where protective effect from having a college education decreased from ARR=0.71 (95% CI 0.53–0.90) to ARR=0.86 (95% CI 0.70–1.06) (Table 4).

**Table 3:** The ARR and 95% CI of children's corporal punishment for the multivariable models without (Model 1) and with (Model 2) the interaction term for women's education level and women's exposure to IPV (n=10,156)

	ARR (95% CI) for children's corporal punishment							
	
	Emotional IPV*		Physical IPV*		Sexual IPV*		Controlling behavior*	
				
Variables	Model 1	Model 2	Model 1	Model 2	Model 1	Model 2	Model 1	Model 2
Emotional IPV	1.17 (1.10–1.25)	0.96 (0.83–1.11)	–	–	–	–	–	–
Physical IPV	–	–	1.15 (1.08–1.22)	1.02 (0.87–1.19)	–	–	–	–
Sexual IPV	–	–	–	–	1.10 (1.02–1.19)	1.02 (0.83–1.26)	–	–
Controlling behavior	–	–	–	–	–	–	1.14 (1.07–1.21)	0.95 (0.81–1.10)
Women's education								
No education	1.00	1.00	1.00	1.00	1.00	1.00	1.00	1.00
Primary school	0.95 (0.89–1.02)	0.57 (0.38–0.85)	0.94 (0.88–1.01)	0.70 (0.47–1.06)	0.95 (0.88–1.01)	0.79 (0.47–1.32)	0.95 (0.89–1.02)	0.59 (0.40–0.88)
High school	0.81 (0.74–0.90)	0.29 (0.13–0.65)	0.81 (0.73–0.90)	0.45 (0.20–1.02)	0.81 (0.73–0.90)	0.56 (0.20–1.55)	0.82 (0.74–0.91)	0.32 (0.14–0.71)
College	0.76 (0.66–0.88)	0.16 (0.05–0.57)	0.78 (0.67–0.91)	0.32 (0.09–1.10)	0.77 (0.66–0.90)	0.45 (0.09–2.07)	0.79 (0.68–0.92)	0.19 (0.06–0.64)
Interaction term	–	1.09 (1.02–1.17)	–	1.05 (0.98–1.13)	–	1.03 (0.93–1.14)	–	1.08 (1.01–1.16)

* Adjusted by women's age (years), residency (urban or rural), socioeconomic status (low, medium-low, intermediate, medium-high, or high), parity, approval of corporal punishment (yes or no), and marital status (partnered or not partnered). Interaction terms equal to the product of women's education and women's exposure to IPV.

ARR=adjusted risk ratios; CI=confidence intervals; IPV=intimate partner violence.

**Table 4:** Relationship between women's education and risk of children's corporal punishment stratified by women's lifetime exposure to emotional IPV and controlling behavior by a partner

	Lifetime exposure to IPV, ARR (95% CI)*			
	
	Emotional IPV		Controlling behavior by partner	
		
Women's education level	No n=5,487	Yes n=4,666	No n=4,846	Yes n=5,317
No education	1.00	1.00	1.00	1.00
Primary school	0.92 (0.83–1.02)	0.97 (0.89–1.06)	0.91 (0.80–1.02)	0.97 (0.89–1.05)
High school	0.72 (0.61–0.85)	0.91 (0.80–1.02)	0.77 (0.66–0.91)	0.85 (0.74–0.97)
College	0.61 (0.47–0.80)	0.98 (0.80–1.19)	0.71 (0.53–0.90)	0.86 (0.70–1.06)

The adjusted risk ratios (ARR) and 95% confidence intervals (CI) are shown.

* All analyses adjusted by women's age (years), residency (urban or rural), socioeconomic status (low, medium-low, intermediate, medium-high, or high), parity, approval of corporal punishment (yes or no), and marital status (partnered or not partnered).

## Discussion

Our main results show that children's CP is common and severe in Nicaragua, and women's exposure to lifetime emotional IPV, physical IPV, sexual IPV, and controlling behavior by a partner increased children's risk of experiencing CP. Our most important finding shows that the protective effect that women's higher education levels had on children's CP fades when women are exposed to lifetime emotional IPV or controlling behavior by a partner.

Our findings show that the prevalence of moderate and severe children's CP in Nicaragua is high and within the range found in other low- and middle-income countries (10–12). The high prevalence of severe forms of children's CP in this setting is an issue of great concern because children's CP has been associated with children's current and future ill-health (1, 2, 4, 6, 7, 11, 13, 44). However, these findings do not come as a surprise because it has been shown that the Nicaraguan society is in general tolerant to children's CP (37), and the Nicaraguan government has failed to pass legal restrictions to ban parents’ use of CP to discipline their children (36).

Our results indicating that all forms of IPV were significantly associated with children's CP are in line with evidence collected from studies around the world (12, 16, 21–23). However, our data also show that the protective effect from women's high educational attainment fades when women are exposed to lifetime emotional IPV and controlling behavior by a partner, two of the most common forms of IPV in Nicaragua (30) and worldwide (45). This is a crucial new finding that demonstrates how significant gains in one positive social determinant of children's well-being (28) can be undermined when it interacts with the violence by men toward women.

Women's education might protect children against CP by providing female caretakers with greater economic resources and by making them less prone to support the use of violence to discipline children (26). However, IPV can significantly increase women's stress and can negatively affect women's physical and mental health (31). Therefore, we argue that this impairment of women's health might reduce the women's ability to take care of their children and might increase the use of CP because of increased frustration and lower tolerance for their children's misbehavior. This proposed pathway is consistent with evidence gathered from our current study and from others indicating that impaired mental or physical health of a caregiver is strongly associated with a higher rate of CP (16–18, 46).

Caregiver approval of physical discipline for children has been shown to be strongly associated with higher children's CP rates (10, 17, 24–26) and positively linked with attitudes tolerating IPV (26). A second possible pathway by which IPV exposure can hinder the protective effect of women's high educational attainment against children's CP is by influencing the caregivers’ attitudes toward CP. A third pathway might involve the men instead of the women in the household. In Nicaragua, men often exhibit hegemonic patterns of masculinity that have high expectations for family obedience to the male head of the household (47). When these demands are not met, abusive men might resort to violence as a way to impose control on women and other family members, including children (48). This suggested pathway is in accordance with the findings in Bancroft and Silverman's study (49) showing that men who are abusive toward their partners are also more prone to slap their children. However, further studies are needed to test this hypothesis.

Our finding that lifetime exposure to only emotional IPV and controlling behavior by a partner significantly interacted with women's educational attainment is puzzling, but not unheard of. A study conducted in Bangladesh found that controlling behavior by a partner was the only IPV exposure associated with higher children's under-five mortality, and only among educated women (50). The authors of this paper suggested that women with higher education might have significantly higher personal autonomy and better attitudes toward gender relations than those with lower education (50). They argue that losing one's autonomy by being exposed to controlling behavior by a partner might be perceived as more damaging to a woman's mental health by those who had high independence levels than by those who had little independence (50). We believe that this argument might also apply to our findings. Emotional IPV is a complex phenomenon that is more universal than physical or sexual IPV, and its effect on women's mental health is as important as the effects caused by other forms of partner violence (51). Because emotional abuse is such a pervasive phenomenon, it is possible that it affects all women regardless of their education level, which is not the case for physical or sexual IPV (45). Clearly, further qualitative studies are needed to explore the complex interactions found in our data.

### Limitations and strengths

Our study uses secondary data that asked the women, not the children, about disciplinary practices in the household. Thus, it is possible that children's CP prevalence in our study is an underestimation of the real figures in the population. In addition, the questions asked in the DHS did not identify the gender of the household members punishing the children. Measuring IPV by including questions in the DHS is challenging, and it might not capture the real magnitude of IPV (52). Consequently, our figures might underestimate IPV exposure in this population. It is important to highlight that the cross-sectional nature of our study does not allow us to establish a causal association between exposures and outcomes. In spite of these limitations, the DHS provides extensive nationwide data on children's CP that allow us to generalize our results to the Nicaraguan population.

## Conclusions and recommendations

This population-based national study shows that the protective effect that women's high educational attainment has on children's CP fades when women are exposed to emotional IPV or controlling behavior by a partner. In its guidelines to prevent child abuse, the WHO has stated that a multi-sectorial approach is needed to curtail violence against children (1). These guidelines suggest the implementation of strategies and actions at all levels of society, and these strategies are more likely to be effective if they also include actions to curtail violence against women, especially IPV. To this end, national policies aiming to end children's CP must include actions that question the societal norms that justify abuse toward children and women and strategies to identify and provide support to women exposed to IPV.
